# Children and adults differ in how primary and secondary incentives modulate valuation, effort, and cognitive control

**DOI:** 10.1371/journal.pone.0351143

**Published:** 2026-06-15

**Authors:** Sebastijan Veselic, Claire Rosalie Smid, Francis Beveridge, Nikolaus Steinbeis

**Affiliations:** 1 Department of Experimental Psychology, University of Oxford, United Kingdom; 2 Division of Psychology and Language Sciences, University College London, United Kingdom; University of Technology Sydney, AUSTRALIA

## Abstract

Rewards have a profound impact on human motivation, cognition, affect, and behaviour. The study of reward processing and incentive effects therefore occupies a central place in psychology and cognitive neuroscience. One common assumption when comparing groups or individuals is that different reward types are valued similarly. Here we examined this assumption in a sample of 51 adults and 39 children (7–12 years) using both primary and secondary rewards. Across three tasks – subjective valuation, willingness to exert cognitive effort, and reward-related modulation of cognitive control – adults showed stronger effects of secondary relative to primary reinforcers, whereas children showed comparatively similar responses across reward types. While we interpret our findings as consistent with age-group differences in the value assigned to secondary reinforcers, larger longitudinal studies using more closely matched incentives will be required to determine how such differences emerge across development. More broadly, our work highlights the importance of carefully considering incentive value when comparing different groups on reward-related processes.

## Introduction

Like many other species, humans are highly sensitive to rewards [1,2]. One core function of rewards is to energize and motivate actions directed towards goals. Rewards therefore play a central role in shaping motivation, cognition, affect, and behaviour, influencing processes as diverse as learning, decision-making, and cognitive control [2–4]. A common assumption in studies of reward processing is that the subjective value of the reward under study is comparable across individuals or groups. This assumption is particularly important in developmental psychology, where many studies implicitly assume that the same incentives are similarly valued across age groups, such that group differences are attributed to changes in the underlying processes of interest rather than to differences in the subjective value assigned to the rewards themselves [5,6]. Here, we compared children and adults to examine whether age-group differences in reward-guided behaviour varied across primary versus secondary reinforcers, using convergent measures spanning explicit ratings, effort allocation, and motivated cognitive control.

Age-related differences in reward-guided behaviour have been documented extensively, including differences in reward sensitivity [7], risk-taking [8,9], intertemporal choice [10–12], prosocial behaviour [13–15], model-based decision-making [16], reward exploration [17], and cognitive control [18]. These behavioural differences are often linked to maturational changes in reward-related neural systems, including developmental changes in dopamine receptor density in striatum and prefrontal cortex [19–22], changes in resting-state connectivity of striatal regions [23], and changes in corticostriatal connectivity from childhood into adulthood [24–28]. For example, adolescents show stronger ventral striatal activation than adults during reward processing even when behaviour is matched across groups [29]. At the same time, recent work suggests that adolescents and adults may differ less in the value they assign to money than in how strategically they allocate effort to obtain it [30]. Although there is little doubt that reward processing changes with age [31,32], some observed age effects may partly reflect the particular incentives used, especially when studies rely heavily on secondary reinforcers such as money; for a few exceptions see [33–35].

The value of secondary reinforcers such as money is acquired through experience [36,37]. It is therefore plausible that their value changes with age. Alternatively, their novelty may make them particularly attractive to younger participants [38]. Under either account, secondary reinforcers may not be valued uniformly across development, which complicates the interpretation of age-group differences in reward-guided behaviour. This is especially relevant given that the goals humans pursue change with development [39], which may shape the types of rewards they value and the degree of value assigned to them [5]. At the same time, preferences for primary reinforcers such as pleasant tastes are also not fixed across development, but are shaped by maturation and exposure [40–42; see also 35]. Thus, if the value of primary and secondary reinforcers changes differently across age groups, then reward-guided behaviour may differ not only because of changes in reward processing itself, but also because of differences in the subjective value of the incentives used.

Previous work investigating similar questions [29,30] focused on adolescents. Here, we focused on middle childhood (7–12 years) because this period is marked by substantial development in executive function, rule use, and motivated behaviour, while also corresponding to increasing everyday exposure to symbolic and other secondary reinforcers, but preceding the broader social and pubertal changes associated with adolescence [5,8,34].

To test whether age-group differences in subjective value and reward-guided behaviour depend on reward type, we directly compared primary reinforcers (pleasant tastes) and age-appropriate secondary reinforcers (money for adults; monetary points exchangeable for prizes for children) [47]. We assessed three aspects of reward-guided behaviour: explicit valuation, willingness to exert cognitive effort, and reward-related modulation of cognitive control. In the present study, we use *valuation* in a pragmatic sense, indexing it through explicit preference reports, willingness to exert cognitive effort, and reward-related modulation of cognitive control. Across the three paradigms, adults showed stronger effects of secondary relative to primary reinforcers, whereas children showed more similar responses across reward types.

## Materials and methods

### Participants

Fifty-one adults (22 male; mean age = 24.61 years, range = 18.7–35.3 years) were recruited through university listings using a subject pool (SONA) with an advertisement indicating that the experiment involved several cognitive tasks performed for rewards. The adult sample primarily comprised undergraduate and graduate students. Thirty-nine children (21 male; mean age = 9.63 years, range = 7.1–12.0 years) were recruited from schools in the Greater London area using an existing lab database and school/community outreach. Parents were initially called and asked whether they would like their child to participate in an experiment with cognitive tasks for rewards. The child sample included 15 White (38.46%), 5 Mixed (12.82%), 2 Black (5.13%), 2 South Asian (5.13%), 1 Southeast Asian (2.6%), 2 participants who would describe their ethnicity as not listed (5.13%), and 12 participants whose parents did not provide information regarding their child’s ethnicity (30.77%) (further details in Table S1 in S1 File). One participant was excluded from all analyses because they failed to respond on most trials. Participants were recruited between 1 February 2019 and 18 December 2022.

Our sample size was comparable to previously published work [43,44], and we increased the number of trials per participant to support robust subject-level estimation of the effects. This included approximately 550 trials per participant in the cognitive control task and approximately 140 trials per participant in the willingness-to-work task. A sensitivity analysis for the achieved sample (children n = 39; adults n = 51; total N = 90) indicated ~80% power at α = .05 to detect a between-group main effect of approximately Cohen’s d ≈ 0.60–0.65 in a two-sample comparison.

Participants were pre-screened for juice-related allergies. All participants provided written informed consent prior to participation. For child participants, written informed consent was obtained from a parent or legal guardian. The study, including all consent procedures, was approved by the UCL Research Ethics Committee in accordance with national regulations (project ID: 12271/003). We report how we determined our sample size, all data exclusions, all manipulations, and all measures in the study.

### Tasks

**Matrix Reasoning**: We administered the Matrix Reasoning subtest from the Wechsler Abbreviated Scale of Intelligence, Second Edition (WASI-II). On each trial, participants viewed an incomplete visual matrix or visual sequence and selected the option that best completed the pattern. Items (30 in total) were presented in order of increasing difficulty, following standard WASI-II administration procedures: at the beginning of administration, three sample items were presented, and administration was discontinued once participants provided three incorrect answers in a row. Furthermore, in line with administration procedures, only items up to number 24 were presented for ages 6–8. Administration typically took approximately five minutes. Thus, before completing the willingness-to-work task, participants had direct experience with multiple matrix items spanning increasing difficulty, although the exact number of items varied across individuals according to standard administration rules.

**Explicit valuation**: In this task, participants were shown either a picture of a juice carton indicating the primary reinforcer or a coin indicating the secondary reinforcer. Crucially, children were told that the coin image corresponds to the points they will be able to earn and exchange for a reward and not actual money. Participants answered three questions on a scale from 1 (least) to 6 (most): “How pleasant do you find this reward?”, “How much would you like to receive this reward?”, and “How happy would you feel right now about this reward?” These questions were modelled on previous work in the field [48]. Each question appeared only once per reinforcer, yielding an overall preference rating independent of reward size.

**Willingness to work:** We developed a novel task for measuring willingness to exert cognitive effort, adapted from prior work on temporal and cognitive effort discounting [49–51]. Participants made binary yes/no choices about whether they were willing to exert varying levels of cognitive effort (4 levels) on the matrix reasoning subtest of the WASI-II in order to obtain rewards that varied in size (6 levels) and type (primary, secondary). Trial combinations were repeated three times in pseudorandom order, resulting in 144 trials, with an additional four familiarization trials at the start of the task. Decisions were not timed, and stimuli remained on screen until a response was made. Participants received a prompt after 2 s if they had not yet responded.

A critical component of the willingness to work task was that effort levels were anchored to each participant’s performance and subjective experience on the matrix reasoning subtest. Because the test is standardized, difficulty could be expressed relative to age-normed performance (t-score distances within an age group). That is, based on participants’ performance and age, we could titrate them into a percentile rank and then use that for determining the cognitive effort levels. These effort levels corresponded to: items solved correctly without effort (“easiest”), items solved correctly but requiring thought (“easy”), items solved with uncertainty (“medium”), and items participants believed they solved incorrectly (“hard”). Participants were introduced to these four effort levels during the instruction phase of the willingness to work task. Note that this “titration process” was not explained to participants. Participants were merely told about the existence of the four cognitive effort levels.

In the willingness to work task, participants were instructed to decide whether they were willing to exert a given amount of cognitive effort for a given reward size. Crucially, they were instructed that their choices would relate to hypothetical items from the matrix reasoning subtest to ensure their responses were not contaminated by their belief about having to provide answers to items that were identical to the ones they had just experienced. They were further told that, from all choices, two would be selected at random at the end of the experiment, one for each reinforcer type. If a “no” trial was selected, they would forfeit any chance of obtaining that reward. If a “yes” trial was selected, they would obtain the reward for a correct answer. If their answer was incorrect, they were told there is a small chance of obtaining the reward (a residual 20% probability). This residual probability was included so that “yes” responses remained meaningful even for higher effort offers where, by definition, they might have considered it impossible to obtain a reward otherwise.

To validate whether participants perceived the four effort levels as increasingly effortful, they completed a control task at the end of the experiment. On each trial, participants saw a cue indicating the upcoming effort level, followed by a matrix reasoning item corresponding to that level. They were given unlimited time to solve the item and then answered questions from the NASA Task Load Index [52]. We used the cognitive load item as an indicator of perceived cognitive effort (range 0–20) and report these ratings in Fig. 3. Importantly, participants did not see the explicit mapping between effort levels and specific items during the willingness-to-work task itself.

**Cognitive control:** We used a standard go/no-go task based on previous work [53,54], with the instruction procedure modelled after [46]. Participants were required to press “f” or “k” depending on the direction of a fish stimulus (left = “f”, right = “k”) or withhold a response depending on stimulus colour (red = withhold response on 33% of trials; blue = respond on 66% of trials). The task combined a go/no-go structure with a congruent flanker-like condition where trial type was cued by fish colour (blue = go; red = no-go) facing the left or right direction. The imbalance in trial frequency was used to induce a prepotent tendency to respond, thereby increasing inhibitory demands on no-go trials. Participants were informed of the colour-response mapping but were not explicitly told the exact go/no-go probabilities. Importantly, reward outcomes depended deterministically on response correctness and reward condition.

The task included three main factors: reward type (primary, secondary), difficulty (easy, difficult), and reward size (low, high) all of which were signalled by visual stimuli. Notably, the visual stimuli used for high and low reward size differed from the visual stimulus indicating reinforcer type. Participants were explicitly instructed how the visual cues mapped onto reward type and reward size and understood these mappings.

On correct trials, adults earned either 2 pence (low) or 6 pence (high) in the secondary-reinforcer condition, whereas children saw the equivalent point increment, which they had been told would later be exchanged for prizes of comparable value. In the primary reinforcer condition, correct responses were rewarded with small squirts of juice delivered using a gustatory stimulation device (0.02 mL for low reward; 0.06 mL for high reward).

Participants first completed 10 familiarization trials, which were also used to initialize an adaptive response threshold. On each trial, participants first saw the condition combination (difficulty and reward size), followed by stimulus presentation. Fish were displayed for 0.4 s on easy trials and 0.2 s on difficult trials. Responses were recorded for up to 1.0 s from stimulus onset. The response threshold used for scoring was updated online as a function of the participant’s recent RT distribution (mean of last 10 trials). On easy trials, 2*SD was added to the mean, on hard trials, 0.5*SD was added. Go responses were scored as correct only if the appropriate keypress occurred before this adaptive threshold; correct keypresses after the deadline were coded as “too slow” and omissions as misses. On no-go trials, withholding a response for the full response window was scored as a correct rejection. Feedback was shown for 1000 ms after each response. Randomized trial sequences were generated for each participant, and there were approximately 550 trials per participant, with around 30 trials per individual condition.

### Procedure

At the beginning of the session, we collected information about participants’ preferred juice flavours (e.g., pineapple, apple, orange), along with self-reported thirst and tiredness. Participants then completed the Matrix Reasoning subtest of the WASI-II [45], which was used to derive age-normed effort levels for the willingness-to-work task.

Participants next completed three tasks under two reward-type conditions (primary and secondary reinforcers): explicit valuation, willingness to work, and cognitive control. Task order and reward-type order were counterbalanced across participants. Instructions were automated and presented on screen, and an experimenter remained present to ensure that participants understood the task requirements. In the child sample, the experimenter conducted verbal comprehension checks before the main tasks began.

Before the tasks, participants were introduced to both reward types. Both groups were told that primary rewards involved receiving varying amounts of their preferred juice. For secondary rewards, adults were told that rewards would be paid out in pounds after the experiment. Children were told that they would earn (monetary) points that could later be exchanged for prizes varying in attractiveness that were comparable in pound value to that of adult rewards. Children were explicitly shown the available prizes. In the child sample, points were used as a secondary reinforcer because money could not be used due to ethical constraints. As a substitute, we chose monetary points because they approximated the key characteristic of money (i.e., allowing goods to be attained). Furthermore, this approach replicated the set-up of previous studies using monetary rewards in children. Notably, for both groups, the visual stimuli they saw related to rewards were identical.

After the three main tasks, participants completed additional measures used to assess possible confounds. These included a need-for-cognition scale, a judgement of the perceived monetary worth of 20 mL of juice, the control task for perceived cognitive effort described above, and post-task preference ratings for low versus high rewards in both the primary and secondary reinforcer conditions.

Adults were asked to avoid drinking fluids for four hours before the experiment in order to standardize thirst levels, following previous work [46,48]. This procedure was not applied to children for ethical reasons. This may have created a difference in “wanting” between the two groups in relation to the primary reinforcer. We therefore collected self-reported thirst measures and repeated key analyses including thirst as a covariate (see S1 File). At the end of the experiment, we also collected data indicating when they had last consumed any liquids.

### Analysis

Data were analyzed using R, version 4.0.0 [55] and the package ggplot2, version 3.3.2 [56]. This study’s design and its analysis were not pre-registered.

**Explicit valuation:** We used a linear mixed-effects model to test whether participants’ valuation of the two reward types differed by group. Subjective value was computed as the mean of the three ratings for each reinforcer. Task order was included as a nuisance covariate because, for some participants, the valuation task occurred after other tasks in which reward contingencies had already been experienced. Owing to technical issues, only one reinforcer condition was recorded for 18 participants. Additional control analyses incorporated perceived monetary worth of juice and self-reported thirst measures (S1 File). We additionally ran a control model that included a further nuisance regressor for the perceived monetary worth of juice and assessed group differences in different aspects of thirst (S1 File).

**Willingness to work**: We quantified effort discounting using an exponential effort-discounting model adapted from prior discounting work [57] using hierarchical Bayesian model fitting in STAN [58]. Briefly, on each trial, the subjective value (SV) of the work option was modelled as:


$$\begin{array}{c} SV_{it}=R_{it}\;\text{exp}\left(-{\left(r_{i}t_{it}\right)}^{s_{i}}\right) \end{array}$$

where $R_{it}$ is reward magnitude, $t_{it}$ is cognitive effort level, $r_{it}$ is the participant-specific effort discounting parameter, and $s_{i}$ is the participant-specific effort sensitivity parameter. Reward magnitude was coded as integer values from 1–6 units. Cognitive effort was coded using t-value distance to define four effort levels: easiest = 1, easy = 7, medium = 13, and hard = 19. A non-zero value was used for the easiest level to allow the model to capture response variability on these trials. Choices were modelled at the trial level with a Bernoulli likelihood ($y_{it}\sim \text{Bernoulli}\left(p_{it}\right)$ logistic choice rule (${\text{p}}_{\text{it}}=\text{P}\left(\text{ch}.\text{ wor}{\text{k}}_{\text{it}}=\text{1}\right)$. The probability of choosing the work option was:


$$\begin{array}{c} P\left(\text{ch}.\text{wor}{\text{k}}_{\text{it}}=\text{1}\right){\text{logit}}^{-\text{1}}\left(\beta_{i}\left(SV_{it}-S\overset{\text{alt}}{\underset{it}{V}}\right)\right) \end{array}$$

where $\beta_{i}$ is a participant-specific choice sensitivity (inverse temperature) and $S\overset{\text{alt}}{\underset{it}{V}}$ denotes the subjective value of the default option. Parameters were estimated using hierarchical Bayesian inference in STAN. Participant-level parameters were drawn from group-level normal distributions on an unconstrained scale and transformed to enforce bounds:


$$\begin{array}{c} r_{i}=\Phi \left(\mu_{r}+\sigma_{r}\overset{\text{raw}}{\underset{i}{r}}\right) \end{array}$$

$$\begin{array}{c} S_{i}=\text{1}\text{0}\Phi \left(\mu_{s}+\sigma_{s}\overset{raw}{\underset{i}{s}}\right) \end{array}$$

$$\begin{array}{c} \beta_{i}=\text{5}\Phi \left(\mu_{\beta }+\sigma_{\beta }\overset{\text{raw}}{\underset{i}{\beta }}\right) \end{array}$$

So that $r_{it}$ ∊ (0, 1), $s_{i}\in \;(\text{0},\text{ 10})$ and β_i_ ∊ (0, 5) with Φ representing the standard normal CDF. We used weakly informative priors:


$$\begin{array}{c} \mu_{r},\mu_{s},\;\mu_{\beta }\sim N(\text{0},\text{1}) \end{array}$$

$$\begin{array}{c} \sigma_{r},\mu_{s},\;\sigma_{\beta }\sim N(\text{0},\text{0}.\text{2}) \end{array}$$

$$\begin{array}{c} \overset{\text{raw}}{\underset{i}{r}},\;\overset{\text{raw}}{\underset{i}{s}},\overset{\text{raw}}{\underset{i}{\beta }}\sim N(\text{0},\text{1}) \end{array}$$

We ran 8 MCMC chains (NUTS) with 10,000 iterations per chain (2,000 warmup; 8 cores). Convergence and sampling quality were assessed using trace plots, $\hat{R}$, effective sample sizes, and checks for divergent transitions. Participant-level parameter estimates used in subsequent statistical analyses were derived from posterior summaries.

We tested whether effort discounting differed by reward type and age group (Group x reinforcer) using mixed-effects modelling on participant-level parameter estimates. In each group, we further used a general linear model to test for an effect of cognitive effort level. This was done to establish that different cognitive effort levels from the willingness to work task were perceived as more cognitively effortful. Because the mapping between cognitive effort level and item was not shown to participants prior to the control task, their responses on the willingness to work task were uncontaminated by these mappings and required an explicit control test.

To assess within-participant reliability in effort-based preferences, we computed a choice-consistency metric defined as the proportion of consistent yes/no responses across the three repetitions of each decision context. This metric provided an index of within-participant choice consistency independent of mean willingness to exert effort.

**Cognitive control:** We used generalized linear mixed-effects modelling (binomial family with logit link) to investigate whether the probability of a correct response in the go/no-go task varied by reinforcer and group. Analyses were conducted on trial-level responses (correct = 1, incorrect = 0) in two separate models: one fitted to go trials and one fitted to no-go trials, allowing us to dissociate reward effects on action initiation (go) from response inhibition (no-go). The fixed effects included group, reinforcer, and their interaction. To account for repeated measures, models included participant-level random intercepts and random slopes for within-subject predictors. In addition, we included random slopes for trial-level nuisance predictors capturing systematic changes across trials (e.g., trial index to model learning and fatigue effects) and response tendencies (e.g., direction biases). The maximal model candidate of interest for our main hypothesis was a response ~ group * reinforcer + (1 + reinforcer + trial_index + direction | sub_id). This was compared to a small set of theoretically motivated candidate models differing in random-effects complexity and nuisance covariates. Models that did not converge or had singular fits were not considered. We performed additional exploratory analyses with more complex models and higher-order interactions that included reward size and difficulty (Tables S2–S6 in S1 File). Due to the limited sample size, these models should be interpreted with caution.

## Results

### Reward-specific valuation differs by age group

**Fig 1:**
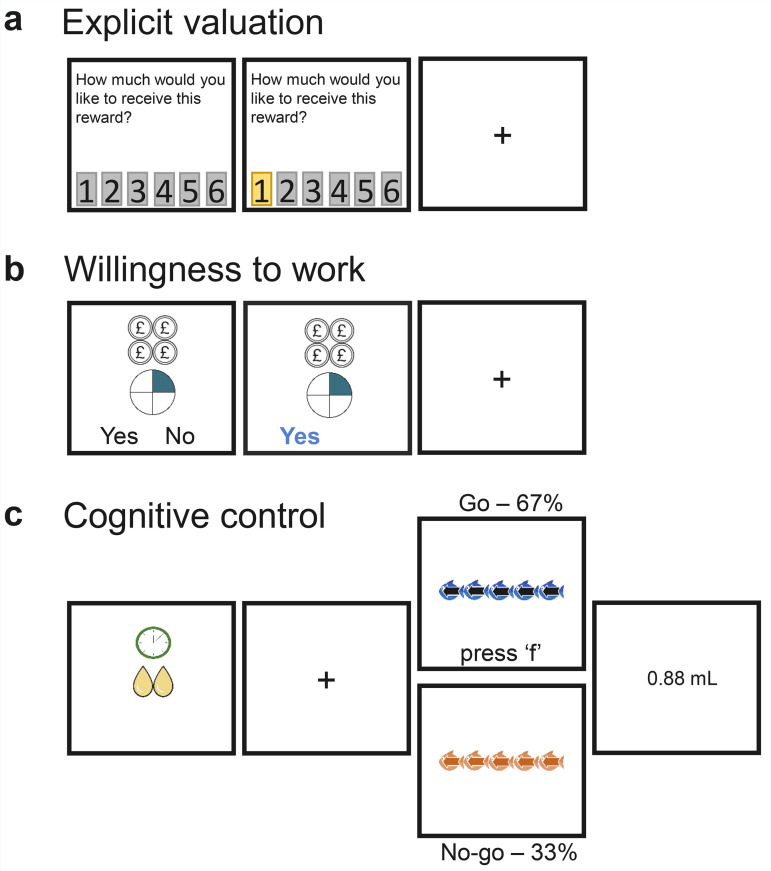
Task descriptions. The experiment consisted of one session and three tasks. **a)** An explicit valuation task (top panel) to establish the subjective value of both reward types. **b)** A willingness to work task (middle panel) to measure the willingness to exert cognitive effort as a function of reward type where reward size (6 levels) and cognitive effort (4 levels) were parametrically varied. **c)** A cognitive control task (bottom panel) to measure the invigorating effect of reward type on cognitive control. In this task, participants were rewarded on a trial-by-trial basis for correct responses either by observing an increase in their cumulative rewards for secondary rewards or by receiving small squirts of juice using a custom-built gustometer for correct responses, as commonly done in rodent or non-human primate research. In addition to reward type, reward size (2 levels), and difficulty (2 levels) were used in this task.

**Fig 2:**
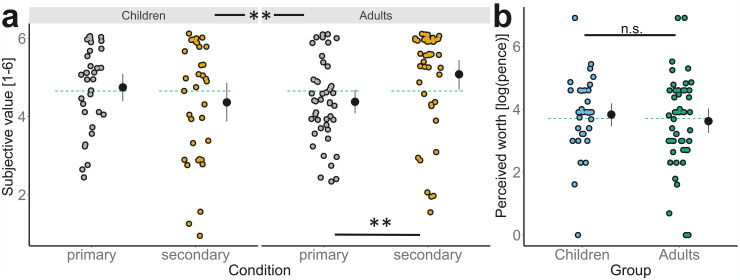
Subjective value of reward differs across age groups. Individual dots depict participants, the dot plot represents the group mean with whiskers representing 95% bootstrapped confidence intervals. The green dotted line represents the grand mean across groups and conditions. **a)** Subjective valuation of reward types in both groups. **b)** Perceived worth of juice in log (pence) across both groups. ** p < .01.

To test our main hypothesis, we first assessed whether participants’ explicit valuation of the two reward types (Fig 1a) interacted with age. We found an interaction (Fig 2a) between group and reward type (χ2 = 7.20, *p* < .01) where adults preferred secondary (*M* = 5.08 ± 0.20) over primary (*M* = 4.38 ± 0.16) rewards (*t*_38_ = 3.15*, 95% CI [0.27, 1.22], p* < .01) whereas children did not (*M*_*secondary*_ = 4.40 ± 0.24, *M*_*primary*_ = 4.75 ± 0.19; *t*_28_ = 0.76, *95% CI [−.41, 0.89]*, *p* = 0.46), with moderate evidence for the null hypothesis (BF_01_ = 3.89). Furthermore, while both groups equally valued the primary reinforcer (*t*_67.57_ = 1.51*, 95% CI [−0.86, 0.12], p* = .14), adults valued the secondary reinforcer more strongly compared to children (*t*_68.02_ = 2.19*, 95% CI [0.06, 1.29], p* = .03). The observed interaction was not explained by group differences in their perception of how much juice is worth in pounds (*t*_82.2_ = 0.76, *p* = 0.45, Fig 2b), with the interaction remaining significant when this information was added as a covariate. Furthermore, this interaction remained significant and was not explained by confounds, such as how thirsty participants were in the beginning of the experiment (see S1 File).

In post-task preference ratings, we further replicated these results (significant Group x Reinforcer interaction (p = .005): adults preferred secondary over primary rewards at both reward sizes (both p < .001), while children showed no preference (both p = ns). Importantly, while both groups preferred large over small rewards (both p < .001), adults rated small secondary rewards more highly compared to children (p < .001).

### Age-group differences in the effect of rewards on cognitive effort are reward-specific

Next, we asked whether group differences in explicit valuation were also reflected in their willingness to exert cognitive effort for such rewards. To test this, we used a novel and age-adapted cognitive effort task in which we parametrically varied reward size (values from 1 to 6 corresponding to one to six images of a given reinforcer) and difficulty level (values from 1 to 4 corresponding to coloured circle quadrants, also denoted as “easiest”, “easy”, “medium”, “hard”, see Fig 1b. See also Methods and S1 File for more details) to examine participants’ willingness to exert cognitive effort.

**Fig 3:**
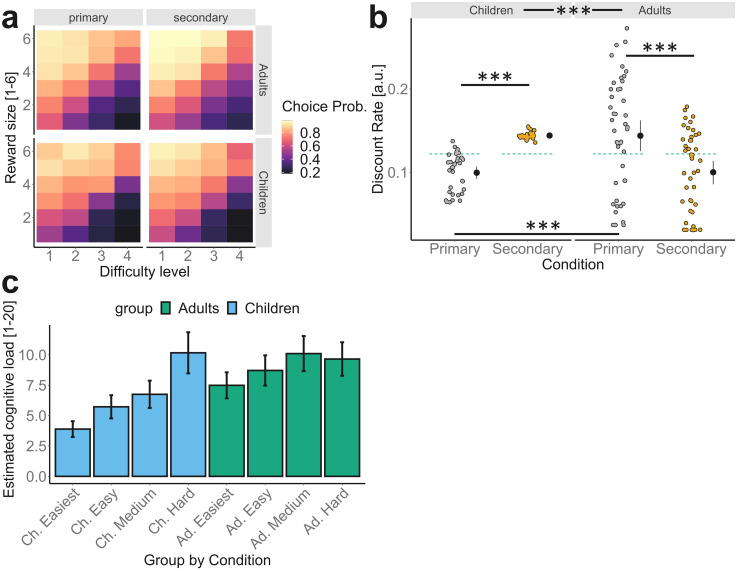
Reward-specific effects on exertion of cognitive effort across age groups. **a)** Average probability of accepting an offer in the cognitive effort task across cognitive effort (i.e., difficulty) levels, reward sizes and reward types for both groups. **b)** Discounting rate (r-parameter) from the cognitive effort model across both groups and conditions. **c)** Estimated cognitive load from the NASA Task Load Index for individual difficulty levels of the control task. *** p < .001.

Participants discounted the value of rewards as a function of cognitive difficulty (Fig 3a) in a similar fashion as shown previously in the domain of physical [61], cognitive [50,59] and time [60] discounting. To quantify and statistically evaluate these differences, we investigated whether participants’ discounting rate (r) parameters for each reward type interacted with age. The parameters were obtained by fitting an effort-sensitive model previously used to model time discounting [57,59,60]. Qualitatively, participants with high r-parameters discount subjective value of a reward more strongly as more cognitive effort must be exerted to receive it, compared to participants with a low r-parameter value. As in the explicit valuation task, we observed a significant interaction between group and reward type on the discounting rate (r) parameter (χ2 = 79.82, p < .001, Fig. 3b). This effect remained after controlling for participants’ choice consistency and self-reported thirst (both p < .001 for the interaction effect, see Methods and S1 File for more details). Adults discounted value more strongly for primary compared to secondary rewards (t_44_ = 4.94, 95% CI [0.026, 0.06], p < .001). However, this difference was reversed in children who discounted value more strongly for the secondary compared to the primary rewards (t_28_ = 10.68, 95% CI [0.04, 0.05], p < .001). Finally, adults more strongly discounted value for primary rewards compared to children (t_56.328_ = 4.03, 95% CI [0.02, 0.07], p < .001). These results demonstrate that adults were more willing to exert cognitive effort for secondary compared to primary rewards, while the opposite was true for children, indicating different types of reward may invigorate both groups when cognitive effort needs to be exerted in cognitive tasks. One interesting pattern observed in children’s discounting rates was reduced variance in the secondary reinforcer condition, suggesting a higher degree of response similarity in the child group as a whole.

As a control, participants were required to rate the experienced cognitive load corresponding to individual cognitive effort levels they were making choices about in the willingness to work task (Fig 1b). To do this, participants were required to solve items of the matrix reasoning subtest at each difficulty level at the end of the experiment. Both groups rated more difficult items as cognitively more effortful on the NASA TLX (children: *F*_(3, 139)_ = 11.46, *p* < .001; adults: *F*_(3, 191)_ = 3.32, *p* = .02, Fig 3c).

### Age-group differences in the invigorating effect of rewards on cognitive control are reward-specific

Finally, we examined whether reward type influenced the allocation of cognitive control differently across groups (Fig 1c). Given that we observed a reward type x group interaction on two separate tasks, one measuring explicit valuation and another willingness to exert cognitive effort, one would further predict a similar reward-type-specific pattern in a task requiring direct allocation of cognitive control [18,28,62–64].

**Fig 4:**
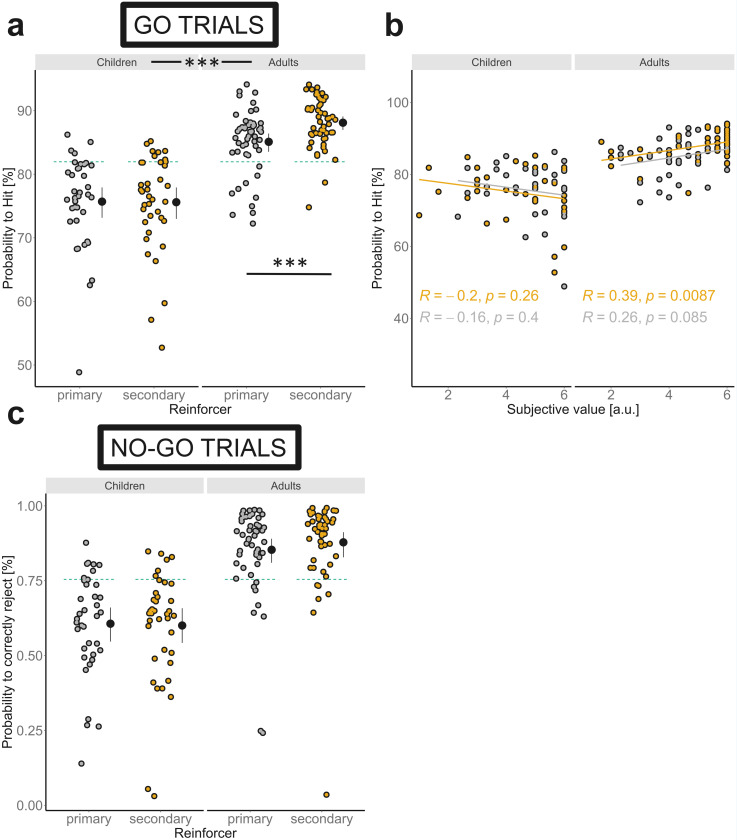
Cognitive control is invigorated in a reward-specific way across age groups. a) Participants’ linear mixed model coefficients across group and reward type on go trials. b) Correlation between participants’ model coefficients and self-reported subjective value for each reinforcer. c) Participants’ linear mixed model coefficients across group and reward type on no-go trials. *** p < .001.

Using generalized linear mixed-effects modelling, we found a similar interaction as in the other two tasks (χ2 = 12.76, p < .001): adults performed better on go trials when rewarded with the secondary compared to the primary reinforcer (t_50_ = 7.79, p < .001, 95% CI [0.02, 0.04]), while children’s performance was comparable across both reward types (t_36_ = 0.27, p = .79; Fig 4a. See also S1 File for additional analyses involving reaction time data, models examining effects of reward size, and confound controls for self-reported thirst), with moderate evidence for the null hypothesis (BF_01_ = 5.47).

Considering the pattern observed on this task resembled the one observed in the explicit valuation task, we predicted the self-reported explicit value ratings would be correlated with their performance estimates on go trials from Fig 4a. Indeed, adults’ subjective value of secondary rewards was correlated with interindividual differences in the go trials for secondary (r = .39, p = .009) but not primary rewards (r = .26, p = .09). In children, these correlations had a reversed sign but were not significant for either the primary (r = −.16, p = .4) or secondary (r = −.2, p = .26) reinforcer. Despite this correlation, we observed no correlation with r-parameters from the willingness to work task (p > .16). This suggests that the pattern across tasks is unlikely to be explained solely by general factors such as greater attentiveness across tasks.

Finally, when we investigated no-go trials for the same effect, we observed no such interaction (χ2 = 0.85, p = 0.36, see Table S4 in S1 File). Notably, Adults were marginally better at inhibiting the prepotent response for secondary compared to primary reinforcers (t_50_ = 1.76, p = .08) while children performed equally well (t_36_ = 0.62, p = 0.54).

## Discussion

Secondary incentives such as money or monetary points are widely used to study motivation and cognitive control across development [10,12,15–18,28,50,65–68,72,73]. Thus, it has been found that compared to adults, children or adolescents take greater risks for secondary reinforcers such as money [72,73], are less generous [67], and more impatient [15,65], while at the same time being less affected by secondary reinforcers in boosting their executive functions [28,66]. These studies are often interpreted under the assumption that such incentives have comparable value across age groups, such that observed differences reflect changes in the process under study rather than differences in the subjective value of the rewards themselves. The present findings challenge that assumption. Across three behavioural tasks: explicit valuation, cognitive effort allocation, and reward-motivated cognitive control, we observed systematic differences between children and adults in how primary and secondary incentives influenced behaviour. Adults showed stronger behavioural modulation for secondary incentives, whereas children responded more similarly for both primary and secondary incentives. These findings are consistent with the possibility that age-group differences in reward-guided behaviour partly reflect differences in the value assigned to the incentives used. However, because of potential differences in motivation and how rewards were introduced, these effects cannot be attributed uniquely to age-related changes in valuation.

The present results relate to an adult literature comparing secondary reinforcers such as money with primary reinforcers such as liquids. Prior work has shown that both types of reinforcers can modulate cognition and behaviour, sometimes to a similar behavioural degree, while relying on partially dissociable neural mechanisms. For example, Beck et al. [46] reported comparable behavioural improvements under monetary and liquid reinforcers during working memory, whereas Yee et al. [74] showed that monetary incentive level and liquid feedback valence can jointly shape motivated cognitive control depending on task structure. In our adult sample, behavioural modulation was stronger under secondary than primary reinforcement, suggesting that the relative impact of different reinforcer types may vary across contexts and populations. Notably, there are clear and genuine reward-related changes through development, as evidenced by structural and functional maturation of reward circuitry [24,26,27]. However, such differences can only be properly understood when behavioural and neural responses are interpreted within the ecology of the developing individual [70].

More broadly, the findings highlight the importance of considering subjective value when interpreting age-group differences in motivated behaviour. Secondary reinforcers such as money acquire value through experience and repeated associations with other desirable outcomes [36,37]. Adults encounter money as a central medium of exchange in daily life, whereas children’s exposure is typically more limited. Reinforcement-learning accounts would therefore predict that secondary reinforcers like money become more influential with repeated experience. This interpretation is corroborated by work showing adolescents aged 12 and above already are invigorated by secondary reinforcers such as money for exerting physical effort [30]. In contrast, the value of pleasant tastes may be shaped differently [2]. Differences in everyday goals and experiences across development may thus alter which incentives are most effective in guiding behaviour and how the relative value difference between them modulates this [5,39]. Our post-task preference ratings support this interpretation: both groups preferred larger over smaller rewards, but the clearest age-group divergence emerged for low-value secondary rewards, which adults preferred more than children. Post-task preference ratings, as used here and in previous work [75], provide a useful way to assess subjective reward value.

One intriguing difference is that we found a reinforcer effect in the cognitive control task only on go trials. We believe this may have arisen for several reasons. First, no-go trials comprised only 1/3 of total trials, which may have limited power to detect more subtle interaction effects. Second, reward may preferentially enhance response initiation or action invigoration rather than inhibitory control itself. Third, because children showed lower overall performance on no-go trials, floor effects may have further reduced sensitivity to detect reward-related differences in inhibition. Our findings also raise a broader question about how the observed differences interact with intrinsic reward [69] or findings showing reward learning remaining stable [71].

Our study has several limitations. First, the study used a cross-sectional design with a modest sample size, which limits our ability to make strong claims about developmental trajectories. While we used a Bayesian approach to estimate evidence for null hypotheses in our data, larger longitudinal samples are needed in future work. Second, the secondary rewards differed across groups: adults received money, whereas children earned monetary points they could exchange for prizes of comparable monetary value. While this allowed us to use developmentally appropriate incentives, it prevented direct comparison of identical reward types across groups. Third, the physiological state differed across groups because adults were asked to avoid fluids before the experiment, whereas children were not. Although the main results remained after controlling for self-reported thirst, this difference remains an interpretational limitation. However, it is worth noting that this may have led to an underestimation of the effects we observed rather than an over-estimation: if children had been subjected to the same procedure, they would have likely rated the primary reinforcer as more valuable. Fourth, we did not calibrate juice volume to individual physiology or track satiety across the session, so group differences in the motivational impact of the primary reinforcer cannot be ruled out (e.g., children may have viewed juice as a treat, whereas adults may have avoided sugary drinks). Relatedly, while the cognitive control task used a brief stimulus presentation window, adaptive response cutoffs meant that there was some degree of individualised calibration also in this task. Fifth, the willingness-to-work task required participants to interpret hypothetical and probabilistic offers; the clustered response patterns observed in children, particularly in the secondary reinforcer condition, may therefore partly reflect simplified strategies or age-group differences in task understanding rather than valuation alone. Sixth, the cognitive control task used brief stimulus presentation and adaptive response deadlines, meaning that age-group differences in processing speed and inhibitory control may have contributed to the observed reward effects. Seventh, the child sample spanned a relatively broad age range but was analysed as a single group. Thus, the present study cannot characterize variation within childhood itself. Finally, demographic comparability across groups was limited because adults were recruited primarily from a university student population, whereas children were recruited through community and school-based outreach.

Despite these limitations, our results show a consistent pattern across three different tasks, including post-task ratings that allowed within-subject assessment of relative value differences across reinforcer types. We believe our work highlights that future studies should carefully calibrate incentives across developmental samples, include reward-free baseline conditions, and consider both motivational and ecological relevance when selecting rewards. Doing so will help ensure that observed behavioural and neural differences reflect genuine differences in reward processing rather than differences in how incentives are valued.

## Supporting information

S1 File Supplementary materials. This file contains the demographic breakdown of child participants and supplementary mixed-model results for go-trial accuracy, go-trial reaction times, no-go-trial accuracy, and reward size and difficulty effects.
(DOCX)

S1 Fig Willingness-to-work choice consistency by group and reinforcer. (PDF)

S2 Fig Post-task preference ratings across adults and children for primary and secondary reinforcers. (PDF)
