# Supplementary material for: Children and adults differ in how primary and secondary incentives modulate valuation, effort, and cognitive control
Source: PLoS One. 2026 Jun 15;21(6):e0351143. doi: 10.1371/journal.pone.0351143 (PMC13268178; doi:10.1371/journal.pone.0351143)
Supplement: S1 File — This file contains the demographic breakdown of child participants and supplementary mixed-model results for go-trial accuracy, go-trial reaction times, no-go-trial accuracy, and reward size and difficulty effects. (DOCX) [file pone.0351143.s001.docx]

**Supplementary Materials**

**Children and adults differ in how primary and secondary incentives modulate valuation, effort, and cognitive control**

# **Supplementary Materials**

| Table S1: Demographic breakdown of child participants | | | |
| --- | --- | --- | --- |
|  | *n* | | *%* |
| Annual Household Income | |  |  |
| £10,000 to £24,999 | 1 | | 2.7 |
| £25,000 to £49,999 | 5 | | 12.82 |
| £50,000 to £74,999 | 4 | | 10.81 |
| £75,000 to £99,999 | 4 | | 10.81 |
| £100,000 or more | 13 | | 35.14 |
| Prefer not to say | 8 | | 21.62 |

## **Group characteristics**

Throughout the experiment, we asked participants a series of control questions that would enable us to rule out confounds driving the observed differences reported in the manuscript.

We tested whether differences in subjective value could have arisen because adults or children were thirstier, wanted to drink juice more, and/or craved it more than the other group. We controlled for these factors by adapting questions from previous work. We observed that adults reported being thirstier (t_56.58_ = 5.10, 95% CI [16.80, 38.51], p < .001), wanting to drink more (t_64.91_ = 3.43, 95% CI [7.66, 29.05], p = .001), and craving juice more (t_72.41_ = 3.67, 95% CI [9.31, 31.43], p < .001) than children. This was expected, as adults were told not to drink for 4 h before the experiment, whereas this was not possible with children for ethical reasons. However, it is unlikely that this difference affected the observed results, as we would have expected the opposite pattern. That is, if this had been a strong determinant of behaviour, adults would have preferred the primary reinforcer over the secondary reinforcer more than children, which was not the case in any of the tasks measured.

## **Willingness to work (WtW) supplementary task explanation**

A critical component of the willingness to work task was the participants’ instructions. Participants were instructed that they had a low chance of attaining a reward ($R$) on any trial on which they would choose “yes”. This was done because the expected value ($EV$) for trials with the highest cognitive effort level was near zero. Namely, the probability of providing a correct answer would be at chance level $P_{chance}$, i.e.

$$EV=RP_{chance}$$

Therefore, participants were instructed that even for incorrect answers there was an additional “low” probability of receiving a reward ($P_{low})$, independent of their performance:

$$EV= R(P_{chance}+P_{low})$$

This incentivized participants to select “yes”, unless they were not willing to exert cognitive effort for a particular trial combination.

## **WtW control results**

We compared both groups on the need for cognition scale because we wanted to ensure that one group would not be inherently more willing to exert cognitive effort because they would find the exertion of cognitive effort rewarding in itself. We found no difference between both groups on the need for cognition scale (M_adults_ = 3.54, M_children_ = 3.33, *t*_73.9_ = 1.80, *95% CI [-0.02, 0.44],* *p* = 0.08). In additional analyses, we added thirst as a covariate to confirm the interaction we observed was not related to differences in participants’ thirst levels. Our results remain significant after thirst is added as a covariate ($\chi2$ = 24.19, p < .001).

In additional analyses, we computed participants’ choice consistency for identical choice option pairs. This analysis revealed that both children and adults are highly consistent in their choices (M_Adults_ = 0.94±0.01, M_Children_ = 0.91±0.007). However, adults were slightly more stable in their responses compared to children across both reinforcers (*t*_Primary_ = 2.28, *p* = .03, *t*_Secondary_ = 2.26, *p* = .03).

Fig S1. Willingness to work choice consistency for each group and reinforcer (see Methods for details on calculation).

## **Cognitive control supplementary results**

We examined whether the effects observed on participants’ probability of providing correct responses would also be observed in log reaction times using linear mixed models, but did not observe an interaction between group and reward (p = 0.43). However, we did find a main effect for both group ($\chi2$ = 6.46, p = 0.01) and reward ($\chi2$ = 29.26, p < .001) (see Table S3).

We also analysed juice delivery volumes across the session, yielding modest volumes (M_adults_ = 87.8 mL ± 2.18, M_children_ = 69.25 ± 2.66 mL). In additional analyses, we added thirst as a covariate to confirm that the interaction we observed was not related to differences in participants’ thirst levels. Our results remained significant after thirst was added as a covariate ($\chi2$ = 64.07, p < .001).

Table S2: Results of generalized linear mixed modelling of participants’ probability to provide a correct answer on go trials.

| ***Fixed Effects*** | *Odds Ratios* | *CI* | *z-value* | *p* |
| --- | --- | --- | --- | --- |
| (Intercept) | 6.08 | 5.34–6.92 | 27.35 | **<0.001** |
| Group | 0.54 | 0.44–0.66 | 6.16 | **<0.001** |
| Reinforcer | 1.32 | 1.18–1.48 | 4.79 | **<0.001** |
| Group * Reinforcer | 0.75 | 0.64–0.88 | 3.57 | **<0.001** |
| **Random Effects** | | | | |
| σ^2^ | 3.29 | | | |
| τ_00_ participant | 0.23 | | | |
| τ_11_ _Participant direction bias_ | 0.30 | | | |
| τ_11_ _Participant Trial effect_ | 0.02 | | | |
| ρ_01_ | -0.56 | | | |
|  | 0.02 | | | |
| ICC | 0.06 | | | |
| N _sub_id_ | 89 | | | |
| Observations | 31924 | | | |
| Marg. R^2^ / Cond. R^2^ | 0.041 / 0.103 | | | |

Table S3: Results of linear mixed modelling of participants’ log reaction times on go trials.

|  |  | | | |
| --- | --- | --- | --- | --- |
| ***Fixed Effects*** | *Estimates* | *CI* | *t-value* | *p* |
| (Intercept) | 5.93 | 5.89–5.97 | 315.16 | **<0.001** |
| Group | 0.07 | 0.02–0.13 | 2.54 | **0.013** |
| Reinforcer | -0.04 | -0.06–-0.03 | 5.41 | **<0.001** |
| Group * Reinforcer | -0.01 | -0.03–0.01 | 0.79 | 0.432 |
| **Random Effects** | | | | |
| σ^2^ | 0.03 | | | |
| τ_00_ _Participant_ | 0.02 | | | |
| τ_11_ _Participant direction bias_ | 0.00 | | | |
| τ_11_ _Participant Reinforcer effect_ | 0.00 | | | |
| τ_11_ _Participant Trial effect_ | 0.00 | | | |
| ρ_01_ | -0.31 | | | |
|  | -0.55 | | | |
|  | -0.05 | | | |
| ICC | 0.37 | | | |
| N _Participant_ | 89 | | | |
| Observations | 31924 | | | |
| Marg. R^2^ / Cond. R^2^ | 0.035 / 0.396 | | | |
|  |  | | | |

Table S4: Results of generalized linear mixed modelling of participants’ probability of correctly withholding the prepotent response on no-go trials.

| **Fixed Effects** | *Odds Ratios* | *CI* | *z-value* | *p* |
| --- | --- | --- | --- | --- |
| (Intercept) | 11.4 | 8.17–15.91 | 14.31 | **<0.001** |
| Group | 0.15 | 0.09–0.24 | 7.71 | **<0.001** |
| Reinforcer | 1.23 | 0.99–1.53 | 1.84 | 0.066 |
| Group * Reinforcer | 0.87 | 0.65–1.17 | 0.92 | 0.358 |
| **Random Effects** | | | | |
| σ^2^ | 3.29 | | | |
| τ_00_ _Participant_ | 1.42 | | | |
| τ_11_ _Participant Trial effect_ | 0.20 | | | |
| ρ_01_ _Participant_ | -0.55 | | | |
| ICC | 0.30 | | | |
| N _Participant_ | 89 | | | |
| Observations | 12485 | | | |
| Marg. R^2^ / Cond. R^2^ | 0.166 / 0.418 | | | |

Table S5: Results of generalized linear mixed modelling of participants’ probability to provide a correct response on go trials.

| **Fixed Effects** | *Odds Ratios* | *CI* | *z-value* | *p* |
| --- | --- | --- | --- | --- |
| (Intercept) | 17.38 | 14.68–20.57 | 33.18 | **<0.001** |
| Group | 0.35 | 0.28–0.45 | 8.58 | **<0.001** |
| Reward size | 0.95 | 0.80–1.12 | 0.63 | 0.527 |
| Difficulty | 0.24 | 0.21–0.27 | 20.12 | **<0.001** |
| Group * Reward size | 1.07 | 0.86–1.33 | 0.64 | 0.523 |
| Group * Difficulty | 1.46 | 1.21–1.76 | 3.94 | **<0.001** |
| Reward * Difficulty | 1.01 | 0.83–1.23 | 0.12 | 0.902 |
| Group * Reward size * Difficulty | 0.84 | 0.65–1.10 | 1.27 | 0.203 |
| **Random Effects** | | | | |
| σ^2^ | 3.29 | | | |
| τ_00_ _Participant_ | 0.27 | | | |
| τ_11_ _Participant Direction bias_ | 0.35 | | | |
| τ_11_ _Participant Trial effect_ | 0.03 | | | |
| ρ_01_ | -0.56 | | | |
|  | 0.16 | | | |
| ICC | 0.07 | | | |
| N _Participant_ | 89 | | | |
| Observations | 31924 | | | |
| Marg. R^2^ / Cond. R^2^ | 0.146 / 0.210 | | | |

Table S6: Results of generalized linear mixed modelling of participants’ probability to correctly withhold a response on no-go trials.

| **Fixed Effects** | *Odds Ratios* | *CI* | z-value | *p* |
| --- | --- | --- | --- | --- |
| (Intercept) | 11.69 | 8.29–16.50 | 13.99 | **<0.001** |
| Group | 0.13 | 0.08–0.21 | 8.15 | **<0.001** |
| Reward size | 1.16 | 0.94–1.43 | 1.36 | 0.175 |
| Difficulty | 1.14 | 0.89–1.45 | 1.04 | 0.300 |
| Group * Reward size | 1.04 | 0.79–1.36 | 0.25 | 0.803 |
| Group * Difficulty | 1.09 | 0.81–1.47 | 0.57 | 0.569 |
| Reward * Difficulty | 0.80 | 0.59–1.08 | 1.44 | 0.149 |
| Group * Reward size * Difficulty | 1.02 | 0.70–1.50 | 0.11 | 0.910 |
| **Random Effects** | | | | |
| σ^2^ | 3.29 | | | |
| τ_00_ _Participant_ | 1.39 | | | |
| τ_11_ _Participant Difficulty effect_ | 0.05 | | | |
| τ_11_ _Participant Trial effect_ | 0.21 | | | |
| ρ_01_ | 0.10 | | | |
|  | -0.53 | | | |
| ICC | 0.31 | | | |
| N _Participant_ | 89 | | | |
| Observations | 12485 | | | |
| Marg. R^2^ / Cond. R^2^ | 0.167 / 0.421 | | | |

## **Post-task preference ratings**

After the experiment, participants rated how much they preferred high and low reward sizes in the primary and secondary reinforcer conditions. Analysing these ratings with a mixed-effects model (random intercepts for participant), we observed a significant group x reinforcer interaction ($\chi2$ = 7.89, p = .005). This replicated the global effect reported for the explicit valuation task. We also found main effects of reward size ($\chi2$ = 31.62, p < .001) and reinforcer ($\chi2$ = 31.62, p < .001).

Fig S2. Preference ratings across adults and children for the secondary and primary reinforcer. After the experiment, both groups rated how much they liked both reinforcer types for each reward size on a scale from 1 (least) to 6 (most). ** p < .01, *** p < .001.
