# Supplementary figures and images for: Children and adults differ in how primary and secondary incentives modulate valuation, effort, and cognitive control
Source: PLoS One. 2026 Jun 15;21(6):e0351143. doi: 10.1371/journal.pone.0351143 (PMC13268178; doi:10.1371/journal.pone.0351143)

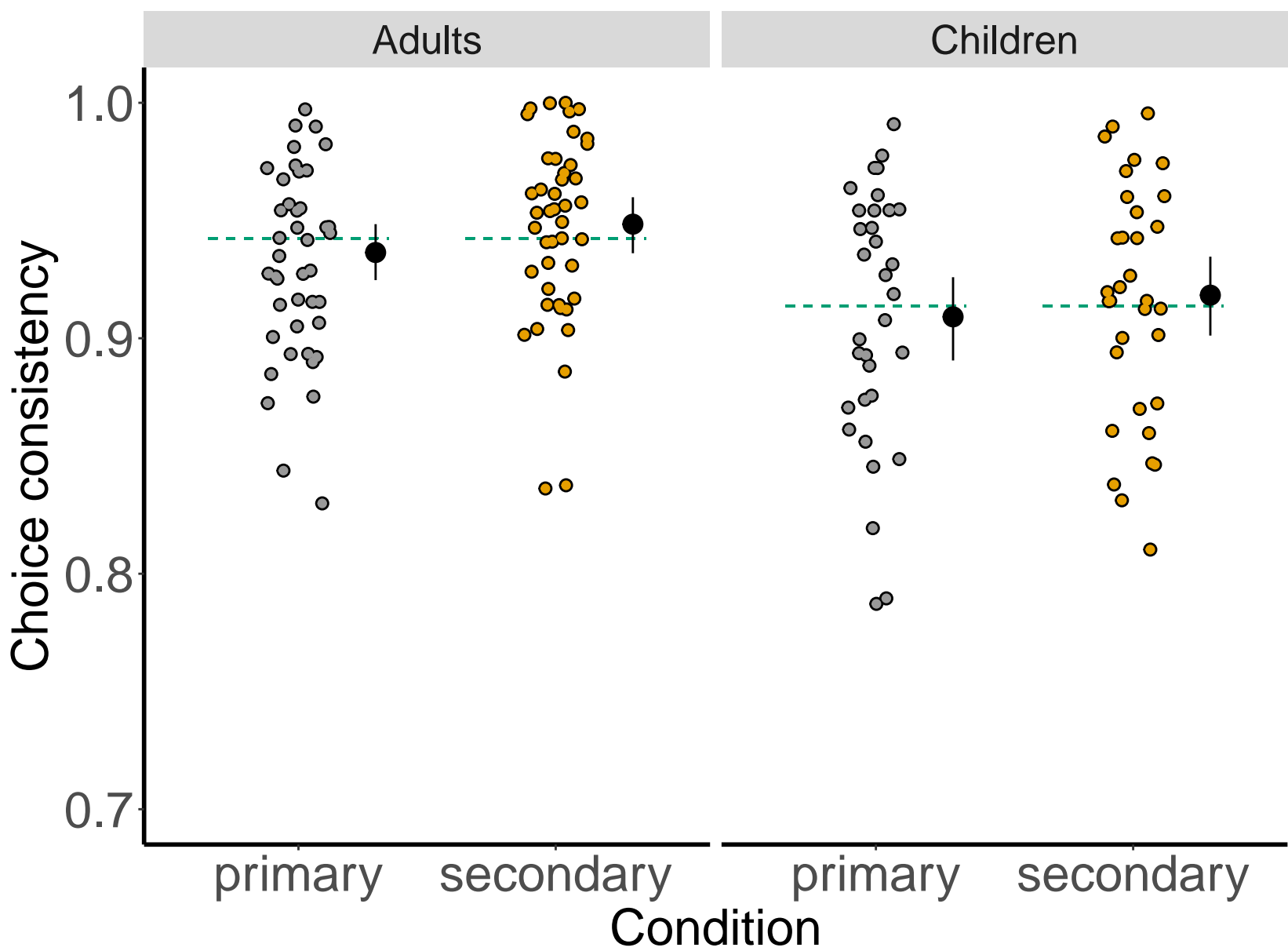

Supplement: S1 Fig — (PDF) [file pone.0351143.s002.pdf]

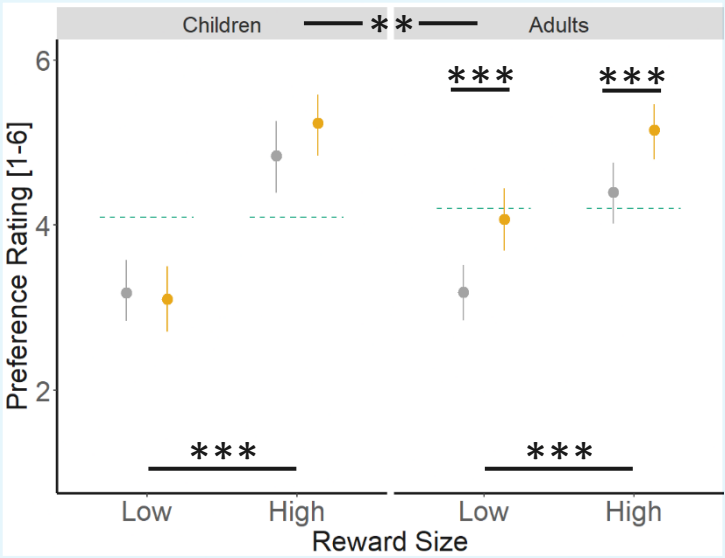

Supplement: S2 Fig — (PDF) [file pone.0351143.s003.pdf]
